# The Role of the Small Ubiquitin-Related Modifier (SUMO) Pathway in Prostate Cancer

**DOI:** 10.3390/biom2020240

**Published:** 2012-04-23

**Authors:** Panagiotis J. Vlachostergios, Christos N. Papandreou

**Affiliations:** Department of Medical Oncology, University of Thessaly School of Medicine, University Hospital of Larissa, Biopolis 41110, Larissa, Greece; Email: cpapandreou@med.uth.gr

**Keywords:** SUMO, SENP, androgen receptor, prostate cancer

## Abstract

SUMO (small ubiquitin-related modifier) conjugation is a reversible three-step process of protein post-translational modifications mediating protein-protein interactions, subcellular compartmentalization and regulation of transcriptional events. Among divergent transcription factors regulated by SUMOylation and deSUMOylation, the androgen receptor (AR) is of exceptional significance, given its established role in prostate carcinogenesis. The enzymes of the SUMO pathway can have diverse effects on AR transcriptional activity, either via direct modification of the AR or through modification of AR co-regulators. Accumulating *in vitro* and *in vivo* evidence implicates the SUMO pathway in AR-dependent signaling. Prostate cancer cell proliferation and hypoxia-induced angiogenesis are also regulated by the SUMO pathway, through an AR-independent mechanism. Thus, an important role has been revealed for members of the SUMO pathway in prostate cancer (PCa) development and progression, offering new therapeutic targets.

## 1. Introduction

Prostate cancer (PCa) is the most common cancer and the second leading cause of death from cancer in males in most western countries. The androgen receptor (AR) is one of the most important factors in PCa development [1]. The transcriptional AR activity modulated by positive or negative regulators plays a critical role in controlling the growth and survival of PCa cells. Enhanced AR activity is essential for cancer cell growth because PCa, in most cases, will regress in response to androgen removal therapy [1,2]. Despite this exquisite sensitivity to androgen deprivation therapy which renders it the most endocrine-sensitive solid neoplasm, advanced disease eventually progresses to castration-resistant PCa. However, recent evidence has shown that cancer progression at this stage is, again, often mediated by AR signaling, so that subsequent AR targeting may further contribute to disease control and, eventually, survival improvement [3]. Given the central role of AR in PCa development and progression, regulators of AR transcriptional activity may have significant effects on these processes.

SUMOylation represents one such important post-translational modification system that regulates the activity of many transcriptional regulators [4]. The continually growing list of SUMO modified proteins comprises various nuclear receptors, including AR as well as transcriptional activators, coactivators, and corepressors [5]. The biological functions of SUMOylation include protein subcellular translocation, subnuclear structure formation, modulation of transcriptional activity, regulation of protein-protein interactions, regulation of cellular metabolism in physiology and disease as well as regulation of several intracellular signaling pathways [6]. SUMOylation depends upon the activity of small ubiquitin-related modifier (SUMO), a protein moiety that is conjugated to a specific lysine residue on target proteins [4]. Three SUMO family members exist, SUMO-1/Smt3C, SUMO-2/Smt3A, and SUMO-3/Smt3B, and all are ubiquitously expressed in mammals [7,8]. At the amino acid level, SUMO-2 and SUMO-3 are 87% identical but only 50% identical to SUMO-1 [7]. Although they exhibit low homology in amino acid sequence, SUMO-1 and ubiquitin are structurally related and share significant similarity in secondary and tertiary structures in higher eukaryotes [9]. Therefore, it is not surprising that the processes of SUMOylation and ubiquitination are mechanistically similar [5]. Like ubiquitination, the conjugation of SUMO is mediated by a series of enzymatic reactions catalyzed by E1, E2, and E3 enzymes that are distinct from those enzymes that catalyze ubiquitination [7]. The SUMO E1 enzymes SAE1 (SUMO-activating enzyme) and SAE2 activate SUMO and transfer it to the E2 enzyme Ubc9, which then directs the conjugated SUMO to its target substrates [7]. Ubc9 then catalyzes formation of an isopeptide bond between SUMO-1 and the ε-amino group of lysine in the target protein. The specificity for the target protein of SUMOylation is thought to reside with Ubc9 itself. SUMOylation typically occurs at a specific sequence, ψKXE, on the target protein where ψ is a hydrophobic residue and X is any amino acid. *In vitro* evidence has indicated that Ubc9 is sufficient for binding to the SUMO acceptor site and efficiently transferring SUMO to selected targets [7]. However, recent evidence shows that a specific E3 ligase might be required for efficient SUMOylation *in vivo*. Three classes of proteins have been identified to have SUMO E3 ligase activity: the protein inhibitor of activated STAT (PIAS) family proteins [10,11], the polycomb protein Pc2 [12], and RanBP2 (Ranbinding protein 2) [13]. The PIAS proteins are reported to act as SUMO-E3 ligases for the SUMO-1 conjugation to AR *in vivo* and *in vitro* [14,15].

SUMOylation is readily reversible in the cell because the isopeptide bond created between the C-terminal glycine in SUMO and the epsilon amino group in the acceptor lysine can be cleaved by SUMO proteases, which are also termed SENPs (SENtrin specific Proteases) or deSUMOylases. There are six SENP enzymes in mammals, each containing a highly conserved 200 amino acid catalytic domain that mediates deSUMOylation [16]. The amino and carboxyl terminal domains vary between SENPs and play roles in subcellular localization and perhaps substrate recognition [17].

SUMOylation and deSUMOylation processes are responsible for transcriptional regulation of various initiated signals, including androgen-mediated transcription. However, there is an established correlation between enhanced androgen-dependence and prostate carcinogenesis and the most striking proof for this is the use of expression of the AR-regulated prostate-specific antigen (PSA) gene as a biologic marker for the diagnosis and treatment of PCa [18,19,20]. The multiple interconnections between members of the SUMO pathway, AR-mediated and AR-independent propagation of PCa are reviewed here, with implications for their ultimate clinical significance.

## 2. Regulation of AR by SUMOylation

The AR can be modified by SUMOylation, preferentially by SUMO-1. Two major SUMOylation sites (K386 and K520) have been identified within the AR [21]. The biological effect of AR SUMOylation was explored by mutating one (or both) SUMOylation sites and measuring androgen-induced transcription. AR containing the Lys 386 to Arg substitution either alone, or together with a Lys 520 to Arg substitution, showed a 2–3-fold enhancement of androgen-dependent transcription on promoters containing multiple androgen response elements (AREs) [21,22,23]. The activity of AR containing the Lys 520 to Arg substitution alone was similar to wild type AR. The data suggest that SUMOylation of AR primarily at Lys 386 reduces the transcriptional activity of AR. The underlying mechanism for this effect has not been defined [17].

Besides SUMOylation, AR is also subjected to phosphorylation and acetylation. Ubc9, the SUMO E2 enzyme, binds the AR within the hinge region [24] that includes the site of direct acetylation of lysine residues at a conserved KLKK motif. However, the SUMOylation of the AR was unaffected by the mutation of the AR acetylation site *in vitro*, probably suggesting that Ubc9 stimulation of AR transcriptional activity may be independent of its ability to catalyze SUMO-1 conjugation [25,26].

AR activity is also regulated by the PIAS family proteins [27]. The PIAS family is composed of several homologous proteins, including PIAS1, PIAS3, PIASxα, PIASxβ, and PIASy. AR-dependent transcription is repressed by PIAS1 and PIASxα in the presence of exogenous SUMO-1 and PIAS RING finger-like domain and enhanced in the absence of SUMOylation. PIAS3 inhibits AR transactivation in LNCaP and HeLa cells but enhances AR activity in HepG2 and AR-overexpressing LNCaP cells [15,28,29,30]. Repression of AR-dependent transcription by PIAS1 and PIASxα is dependent on the ectopic expression of SUMO-1, indicating that the SUMOylation of AR is crucial for AR-dependent transcriptional repression [14]. PIASy-mediated enhancement of AR-dependent transcriptional repression, however, seems to be independent of SUMOylation [31,32].

A novel PIAS-like protein, named hZimp10, was identified as an additional AR co-regulator. hZimp10 co-localized with AR and SUMO-1 at replication foci throughout the S phase, and it was capable of enhancing SUMOylation of AR *in vivo*. Studies using SUMOylation-deficient AR mutants suggested that the augmentation of AR activity by hZimp10 is dependent on the AR SUMOylation. A link was revealed between hZimp10-mediated enhancement of AR SUMOylation and modulation of AR-mediated transcription, although it remains uncertain whether hZimp10 acts directly as an E3 ligase [32,33].

The SUMOylation acceptor sites of the AR have also been mapped to a previously identified inhibitory motif called the synergy control (SC) motif [34]. The SC motif has also been found within the negative regulatory regions of many different transcription factors [34] and their SUMO acceptor sites have been defined within these SC motifs [26]. Mutation of these SUMO acceptor sites in these factors leads to an increase in the transcriptional activation. These findings suggested that SUMOylation-dependent repression is a common regulatory mechanism in transcriptional control [26].

But what is the molecular mechanism through which SUMOylation regulates AR transcriptional potential? It was shown that a conserved motif localized at the hinge region of the AR is responsible for acetylation and that the extent of AR SUMOylation is independent of its acetylation status [35], excluding the possibility of SUMOylation antagonizing the same lysine residues for acetylation. Furthermore, the same research group reported that PIAS family proteins PIAS1 and PIASxα could enhance AR SUMOylation but could not alter the subnuclear localization of the AR [35], indicating that SUMOylation of the AR is irrelevant to AR subnuclear distribution. In addition, it was also demonstrated that SUMOylation does not affect its DNA-binding capability [23].

A mechanistic insight underlying SUMO-dependent transcriptional repression of the AR was provided by the implication of Daxx, initially identified as a cytoplasmic signaling molecule linking Fas receptor to Jun *N*-terminal kinase in Fas-mediated apoptosis [36], in this process. Daxx has recently been demonstrated to act within the nucleus to regulate gene expression, inhibiting the activity of several transcription factors. The finding that Daxx selectively binds to SUMOylated AR *in vitro* and *in vivo*, combined with evidence of suppression of AR transcriptional activity by wild type, but not mutant SUMO, strongly suggest that AR SUMOylation is involved in regulating Daxx-dependent transcriptional repression [37]. Accordingly, mutation of SUMO-conjugated sites in AR resulted in a loss of Daxx interaction and an increase of AR transcriptional activity [26].

The most recent data on SUMO-mediated repression of AR transcriptional activity involves histone deacetylase 4 (HDAC4) binding. Intriguingly, this was found to depend on SUMOylation, rather than deacetylation, of the AR. HDAC4 increases the level of AR SUMOylation in both whole-cell and cell-free assay systems, raising the possibility that the deacetylase may act as an E3 ligase for AR SUMOylation. Knock-down of HDAC4 increases the activity of endogenous AR and androgen induction of PSA expression and PCa cell growth, which is associated with decreased SUMOylation of the receptor. Overall, HDAC4 has emerged as a new positive regulator of AR-mediated transcription, revealing a deacetylase-independent, SUMOylation-dependent mechanism of HDAC action in PCa cells [38].

Further enhancing the diversity of SUMOylation-induced effects on AR signaling, and being at odds with the previously observed negative effect of SUMO-1 conjugation on AR-initiated transcription, SUMO-3 may have a negative or strongly positive effect on AR, depending on the type of PCa cells. In primary prostate epithelial cells, PrEC, and the PCa cells, PC-3, SUMO-3 has a weak negative effect on AR transcriptional activity. In contrast, SUMO-3 and its close relative SUMO-2 strongly enhance transactivation by endogenous AR in LNCaP cells. This positive effect is observed in both androgen-dependent and androgen-independent LNCaP cells. Mutational analysis of AR and SUMO-3 demonstrated that the SUMO-3-mediated transcriptional activation does not depend on either the previously-identified SUMOylation sites of AR or the covalent conjugation of SUMO-3 to target proteins [15]. These results suggest a novel mechanism for elevating AR activity through the switch of SUMO-3 from being a weak negative regulator in normal prostate cells to a strong positive regulator in PCa cells. This further implies that SUMO-2 and SUMO-3 stimulate the proliferation of PCa cells that is independent of AR SUMOylation, making it mechanistically distinct from the SUMO-1-dependent repression of AR activity. SUMO-3 may thus have an important role in promoting prostate carcinogenesis [15].

In addition, strong clinical data regarding immunohistochemical expression of SUMO pathway member proteins in human specimens further support a critical role for protein SUMOylation in PCa development and progression. Endogenous basal PIAS3 expression was reported to localize in the nucleus in a majority of epithelial and endothelial cells. Increased PIAS3 expression was observed in 100/103 samples examined in a variety of human cancers including prostate, lung, breast, colorectal, and brain tumors. Differential PIAS3 expression and the specific patterns might therefore be useful as a molecular tumor marker [39].

Likewise, it was found that in primary PCa, Ubc9 expression is increased compared with normal tissue, whereas in metastatic prostate tissues, it is decreased in comparison with their corresponding normal and primary adenocarcinoma tissues. Ubc9 expression correlates negatively with tumor Gleason score. The authors conclude that expression of Ubc9 is directly associated with progression of PCa, since it was high in prostatic intraepithelial neoplasia (PIN) cells and even higher in primary adenocarcinomas [40]. To explain this, the authors postulate that given that the N-terminal half of the AR hinge region is essential for the interaction with Ubc9, and AR transcription is enhanced by coexpression with Ubc9 [24], it is possible that Ubc9 contributes to PCa progression by modulating AR stability and/or activation. A correlation between level of Ubc9 expression and the presence/extent of host-immune infiltrate in primary PCa was another key finding of this Ubc9-based TMA analysis [40].

## 3. Regulation of AR by deSUMOylation

AR SUMOylation is a dynamic process and is reversed by SENP1, which promotes AR-dependent transcription in PCa cells. Nucleocytoplasmic shuttling is an important parameter that should be always considered in SUMOylation and deSUMOylation events related to AR. Nuclear import of AR is not sufficient for SUMOylation, because constitutively nuclear apo-ARs or antagonist-bound ARs are only very weakly modified by SUMO-1 in comparison with agonist-bound ARs. Of the SUMO-specific proteases (SENP)-1, -2, -3, -5, and -6, only SENP1 and SENP2 are efficient in cleaving AR-SUMO-1 conjugates in intact cells and *in vitro*. Both SENP1 and -2 are nuclear and found at sites proximal to AR. Their expression promotes AR-dependent transcription, but in a promoter-selective fashion. SENP1 and -2 stimulated the activity of holo-AR on compound ARE-containing promoters. The effects of SENP1 and -2 on AR-dependent transcription were dependent on catalytic activity and required intact SUMO acceptor sites in AR, indicating that their coactivating effects are mainly due to their direct isopeptidase activity on holo-AR. In PCa cells, ectopic expression of SENP1, but not that of SENP2, increased the transcription activity of endogenous AR. Silencing of SENP1 attenuated the expression of several AR target genes and blunted androgen-stimulated growth of LNCaP cells. These results indicate that SENP1 reverses AR SUMOylation and helps fine-tune the cellular responses to androgens in a target promoter-selective manner [22]. Intriguingly, SENP1’s ability to enhance AR-dependent transcription is not mediated through deSUMOylation of AR, but rather through its ability to deconjugate histone deacetylase 1 (HDAC1). Thus, SENP1-dependent deSUMOylation of HDAC1 reduces its deacetylase activity and repressive activity to AR-dependent transcription [41,42,43].

SENP1 levels were enhanced in cells treated with IL-6, which is a positive regulator of the activation of AR-dependent transcription through the activation of the MAPK and JAK/STAT pathways [44,45]. The combination of both a synthetic androgen (R1881) and IL-6 profoundly enhanced SENP1 expression by more than seven-fold, compared to either compound alone, and this was further supported by an enhanced production of PSA. Conversely, inhibition of SENP1 expression by siRNA reduces androgen-induced PSA production in LNCaP cells [46]. Thus, it is likely that SENP1 induction is essential for androgens and IL-6 to induce PSA secretion.

These *in vitro* findings translate to an altered clinical phenotype, depending on the levels of SENP1 expression, as evidenced by induction of PIN-like structure formation in SENP1 transgenic mice that were older than 4 months [2]. Elevated SENP1 expression has also been detected in human PCa at the PIN stage [20]. SENP1 messenger RNA was increased in 29 of 43 cases of high grade PIN (67%). Similarly, SENP1 expression was increased in 26 of 43 PCa samples (60%). Thus, SENP1 expression is preferentially increased during the development of PCa in the majority of cases. Collectively, these studies indicate that overexpression of SENP1 is likely to play a significant role in PCa development [20].

## 4. Androgen-Mediated Stimulation of AR SUMOylation and deSUMOylation

Androgen treatment of PCa cells stimulates AR SUMOylation within 15 minutes and reaches a maximum level by 1 hour [22]. The similarity between these kinetics and androgen-induced nuclear import of AR, and the fact that SUMOylation enzymes E1 and E2 are highly concentrated in the nucleus, suggested that AR SUMOylation might occur only in the nucleus after nuclear import. However, this is not the case, based on the observation that AR can be SUMOylated in either the cytoplasm or nucleus, with similar efficiencies [22]. The androgen-mediated induction of AR SUMOylation could therefore reflect a conformational change that enhances accessibility of the modification site or the interaction of AR with SUMOylation enzymes. Under steady state conditions, only a small fraction of the total AR pool is conjugated with SUMO [22]. 

Investigation of the protein expression profile of mouse prostate by the use of subcellular fractionation, 1-DE (one-dimensional gel electrophoresis) protein separation and mass spectrometry, also demonstrated that both free SUMO-2/3 and SUMO-1 are particularly abundant in the prostate and their levels are subject to tight control by the androgen 5R-dihydrotestosterone (DHT) [47].

DeSUMOylation is also subject to tight control by androgens, as exposure of LNCaP cells to androgen treatment enhances SENP1 transcription. This androgen-mediated augmentation of SENP1 is an AR-dependent event, as reflected by the absence of the effect both when the androgen receptor antagonist bicalutamide is present, and in AR-negative PCa PC-3 cells. A specific ARE on the promoter of the SENP1 gene is required for SENP1 induction by androgens [48]. Thus, elevation in SENP1 mRNA levels in PCa cells is selective and requires the activation of the AR. Upon activation, AR binds this ARE and activates SENP1 transcription. Therefore, SENP1 regulates androgen-AR signaling through a positive feedback mechanism, as follows: The androgen-activated AR binds a specific response element located proximal to the SENP1 promoter. SENP1 promoter activity is enhanced by this activated AR, and thereby, SENP1 mRNA levels are significantly elevated. SENP1 up-regulation completes the positive feedback loop by potentiating AR-dependent transcription and cell proliferation [49]. 

It is intriguing to speculate that androgen ablation therapy is initially effective in treating PCa due to its ability to decrease SENP1 expression. It could therefore be hypothesized that therapeutic agents designed to selectively lower SENP1 levels might be more effective than androgen ablation therapy in the treatment of advanced PCa. Like androgen ablation, reduction of elevated SENP1 levels lowers AR activity [49,50] and PCa cell proliferation. However, unlike androgen ablation, selective down-regulation of SENP1 could modulate these two events without depleting prostate epithelial cells of androgen. By not altering androgen levels, this SENP1-targeting agent would not prompt the development of androgen-dependent cancer cells [49].

## 5. Indirect Regulation of AR-Dependent Transcription by the SUMO Pathway

Emerging data implicate SUMOylation in regulating AR activity by modulating co-regulator activity. Two such factors include p68 (DDX5) and p72 (DDX17), which are members of the DEAD-box RNA helicase family that can unwind double stranded RNA and contribute to the remodeling of ribonucleoprotein complexes. These activities of p68/p72 are required for efficient RNA splicing and microRNA processing. p68/p72 perform functions that are independent of their helicase activity. This is especially common in their role as transcriptional coactivators, where p68/p72 regulate various transcription factors, including AR [50]. One conserved lysine residue in the N-terminus of p68 and p72 RNA helicase (K53 and K50, respectively) has been identified to be a target for SUMOylation [51,52,53]. SUMOylation of p68/p72 has a variety of consequences: It doubles protein stability of p68, whereas it only slightly increases the protein half-life of p72. This may translate into a significant clinical effect, as p68 was found to be overexpressed in prostate tumors and is capable of coactivating AR [54], the key player in prostate tumorigenesis, and could thereby facilitate prostate tumorigenesis [50].

Alien thyroid hormone receptor-interacting protein (TRIP15)/subunit 2 of the signalosome complex (CSN2) binds to the amino-terminus of AR with the receptor SUMOylation sites being involved. Both the AR N terminus with the first 328 aa and the SUMOylation sites are involved in the regulation of the Alien-AR interaction, as deletion of the inhibitory domain or mutating the SUMOylation sites of AR leads to an increase of AR-mediated transactivation. Thus, SUMOylation might have a possible role for binding to Alien, although Alien binds non-SUMOylated AR at least *in vitro*, suggesting the notion that SUMOylation *per se* is not involved for binding of Alien to AR [55]. Alien has characteristics of a co-repressor as it is recruited to AR in the presence of the AR antagonist cyproterone acetate (CPA). Furthermore, cellular localization of Alien is changed towards a predominant nuclear localization upon treatment of PCa cells with CPA. Notably, stable expression of Alien in LNCaP cells inhibits both endogenous PSA expression and proliferation of these cells in the presence of CPA, but not in the presence of an AR agonist. These findings underline the importance of co-repressors for inhibition of PCa cell growth by androgen antagonists [55].

A multi-C2H2-type zinc finger protein (ZNF), ZNF451, was found to interact with both the SUMO E2 conjugase Ubc9 and SUMO-1, SUMO-2 isoforms. Further, ZNF451 interacts in a SUMO-1-enhanced fashion with AR. Although it lacks an intrinsic transcription activation function, ablation of endogenous ZNF451 in LNCaP PC cells significantly decreases expression of several AR target genes. Thus, ZNF451 acts as a transcriptional co-regulator. Both ZNF451 and AR are expressed in PCa cells. SUMOylation status of ZNF451 determines its subnuclear localization, suggesting that movable ZNF451 may influence transcription by modulating SUMO-directed trafficking of co-activator or co-repressor proteins between nucleoplasm and nuclear bodies (NBs). It is of note that AR itself evades NBs, and therefore, a “courier,” such as ZNF451, is likely to be involved in the release of co-activators from nuclear storage sites. Furthermore, ZNF451 may also tether transcriptional co-repressors, such as HDAC4, and thereby relieve repression: that is, de-repress AR activity. These data characterize ZNF451 as a novel SUMO-associated co-regulator protein that regulates androgen signaling [56].

Pontin, a component of chromatin-remodeling complexes, is also SUMO-modified, and SUMOylation of pontin is an active control mechanism for the transcriptional regulation of pontin on AR target genes in PCa cells. Biochemical purification of pontin-containing complexes revealed the presence of the Ubc9 SUMO-conjugating enzyme that underlies its function as an activator. Intriguingly, DHT treatment significantly increased the SUMOylation of pontin, and SUMOylated pontin showed further activation of transcription of a subset of nuclear receptor-dependent target genes and led to an increase in proliferation and growth of PCa cells. These data clearly define a functional model and provide another link between SUMO modification and PCa progression. It would therefore be tempting to explore the possibility that malignant progression of PCa cells might be dependent on SUMOylation of pontin [57].

**Figure 1:**
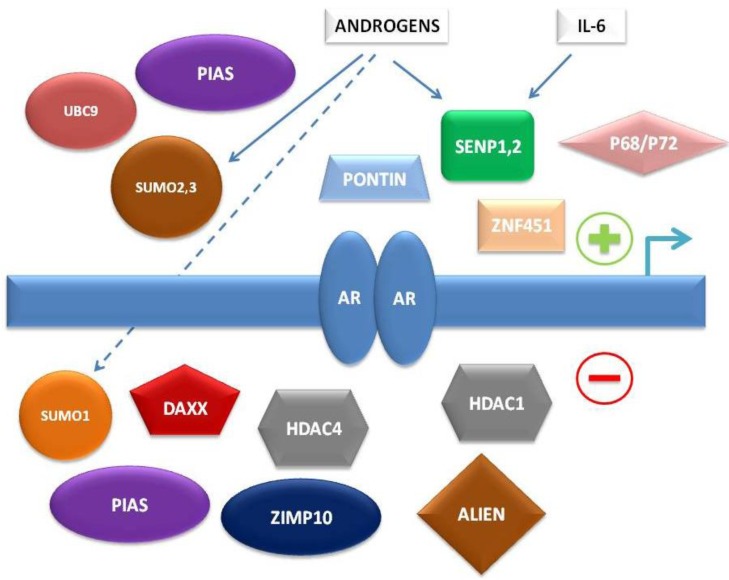
Simplified schema of interconnections between small ubiquitin-related modifier (SUMO) pathway proteins and other factors regulating androgen receptor (AR)-dependent transcription.

To summarize, multiple levels of regulation of AR-dependent transcription by the SUMO pathway have been characterized in recent years, involving either direct interaction of SUMO pathway member proteins with AR or indirect control of AR-associated co-regulators (Figure 1). 

## 6. AR-Independent Regulation of PCa Growth by the SUMO Pathway

SUMOylation regulates the activity of prostate carcinogenesis through a variety of mechanisms, some of which are unrelated to AR signaling. For example, the Sp family of transcription factors, which contains three proteins, Sp3, M1 and M2, with differing capacities to stimulate or repress transcription. This was demonstrated in a series of experiments monitoring the activity of the natural promoter of PSA in its natural cellular milieu (prostate epithelial cells) in conjunction with expression vectors encoding wild-type and point-mutated Sp proteins. A yeast two-hybrid screen to identify Sp3-binding proteins resulted in the identification of Ubc9 as an M2-binding protein, and sequence analyses identified consensus SUMOylation motifs within several Sp family members. Western blots probed with anti-Sp3 detected a high molecular weight Sp3 isoform that is stabilized by a SUMO-1 hydrolase inhibitor, and this protein is also bound by anti-SUMO-1 antiserum. Transient transfection assays with epitope-tagged-SUMO-1 and GFP-SUMO-1 fusion proteins confirmed that Sp3, M1 and M2 proteins are SUMOylated *in vivo*. Substitution of arginine for lysine at one putative site of SUMOylation, lysine551, blocked SUMOylation of all Sp3 isoforms *in vivo* and led to a marginal increase in Sp3-mediated transactivation in insect and mammalian cells. In contrast, introduction of this amino acid substitution within M1 converted it into a potent transcriptional activator. Therefore, Sp3 isoforms are SUMOylated *in vivo* and this post-translational modification plays an important role in the regulation of Sp3-mediated transcription. Although SUMOylation appears to only modestly reduce trans-activation by Sp3, SUMOylation of M1, and presumably M2, is required for a mechanism of transcriptional repression that is insensitive to HDAC inhibition (by trichostatin A). Regardless of the precise mechanism by which SUMOylation converts M1 and M2 into transcriptional repressors, it is evident that Sp3 isoforms are differentially regulated by SUMOylation, and the abundance of SUMOylated M1/M2 proteins determines overall levels of Sp3-mediated transcription [58].

Provided SENP1 can strongly increase AR transcriptional activity, an effect of SENP1 on proliferation was demonstrated in androgen-dependent PCa, as endogenous SENP1 silencing by SENP1 siRNA in LNCaP cells restricted cell growth. However, similar results were observed in PC-3, an androgen-independent PCa cell line, suggesting that SENP1 might play a role in regulating cell proliferation through an AR-independent pathway. Also, the number of SENP1-silenced PC-3 cells in the G1 phase was significantly increased but was decreased in the S and G2/M phases, suggesting that SENP1 may promote G1–S phase transition in PC-3 cells. This slowing of the proliferation of SENP1-silenced PC-3 cells may thus be due to an alteration in G1–S phase progression. Silencing SENP1 expression decreased the expression of cyclin D1, but not cyclin E, in PC-3 cells. Conversely, stably transfected SENP1 in LNCaP cells enhanced cyclin D1 expression and cell proliferation. Interestingly, cell proliferation is dependent on cyclin D1 expression induced by SENP1. Thus, the regulation of cyclin D1 expression by SENP1 is another means through which PCa cell growth is regulated. The induction of cyclin D1 expression by SENP1 depended on its catalytic activity, as the mutation of SENP1 catalytic domain disrupted its activity on cyclin D1 transcription and cell proliferation [2].

Hypoxia-inducible factor (HIF) 1α stabilization, enhanced vascular endothelial growth factor (VEGF) production, and angiogenesis is regulated by SENP1 during prostate carcinogenesis. In the absence of SENP1, HIF1α is actively SUMOylated and subsequently degraded under hypoxic conditions [1,2]. SENP1 alters VEGF levels by directly regulating HIF1α stability during fetal development [1,2]. Furthermore, SENP1 transgenic mice exhibited increased expression of HIF1α with progression of the dysplasia. The enhanced HIF1α stability in the SENP1 overexpressing mice produced elevated VEGF expression. Consequently, it is not surprising that angiogenesis was readily observed in these SENP1 transgenic mice compared with age-matched wild-type mice. In two lines of SENP1 transgenic mice, the hyperplasia further progressed to develop PIN [2]. Also, high-grade PIN was observed in the transgenic mouse line with the higher level of the SENP1 transgene. Enhanced proliferation of prostate epithelia was observed in the SENP1-overexpressing mice, and concurrently, pro-oncogenic factors: specifically the androgen receptor (AR) and cyclin D1, were elevated [2]. Thus, SENP1 participates in the development of prostate neoplasia via facilitating both pro-growth and angiogenic pathways. In the well-defined PCa mouse model TRAMP, high-grade PIN is accompanied by an increase in HIF1α levels, which is, in turn, required for initiation of the angiogenic switch [59]. Consistently, SENP1 transgenic mice exhibit an induction of HIF1α with the initial onset of PIN (or low-grade PIN) at 4 months of age, suggesting that SENP1 regulation of HIF1α occurs early in prostate pathogenesis. Also, SENP1 overexpression initiates the HIF1α pathway in the prostates of transgenic mice as indicated by the elevation of HIF1-regulated VEGF protein levels at low-grade PIN (4 months of age) and even more dramatic VEGF elevation at 12 months of age, when the SENP1 transgenic mice concurrently exhibit an increase in microvessel density [2]. Given that HIF1α is currently being evaluated as a prognostic marker for PCa aggressiveness, it is intriguing to speculate that because SENP1 modulates HIF1, SENP1 may be an equally good marker. This fosters the need for more comprehensive studies to evaluate the potential of SENP1 as a prognostic marker in human PCa [2]. Intriguingly, HIF1 has also been demonstrated to be SUMO-1 modified even in the presence of SENP-1. Work by Berta *et al*., 2007 reported that when HIF1α is conjugated to SUMO, its transcriptional activity is decreased and that this is not mediated by a change in the protein's half-life [60]. Most importantly, HIF1α also regulates SENP1 as a transcriptional factor, thus contributing to formation of a positive feedback loop which is important in VEGF production, essential for angiogenesis in endothelial cells [61].

**Figure 2:**
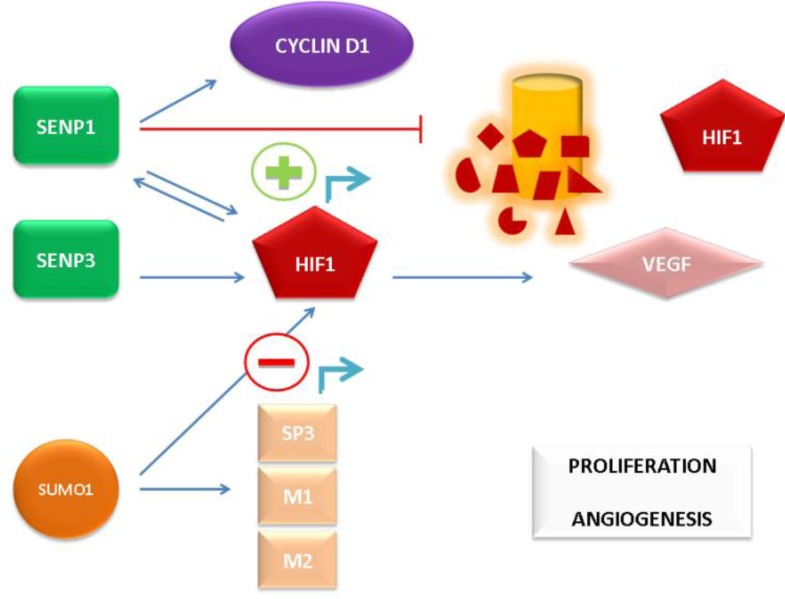
AR-independent promotion of prostate cancer (PCa) cell proliferation and angiogenesis by the SUMO pathway.

Assessment of tissue from human PCa patients indicates elevated mRNA levels of both SENP1 and the SUMO-2/3 deconjugating enzyme, SENP3. SENP3 regulates the transcriptional activity of HIF1α via deSUMOylation of the coregulatory protein p300. Through this mechanism, overexpression of SENP3 facilitates the expression of HIF1α-regulated VEGF, which is critical for vascular development. Induction of SENP3 can be mediated via reactive oxygen species (ROS), as the latter can inhibit the ubiquitin-proteosomal mediated degradation of SENP3 to increase SENP3 protein levels [62,63]. Hence, the induction of SENP3 directly contributes to cancer progression, providing another attractive alternative target for therapy, possibly most notably in cancers with increased ROS levels, including PCa [63,64]. The aforementioned mechanisms of SUMO pathway-mediated AR-independent induction of PCa growth and angiogenic signaling are depicted in Figure 2. 

Progression of PCa to androgen-independent growth involves many oncogenic signaling pathways, some of which are regulated by SUMOylation, as described above. Furthermore, since aberrant AR signaling (regulation of which is intimately associated with the SUMO pathway) is an important driving force of hormone resistance in PCa, the impact of the SUMO pathway may play a role in the development of androgen-refractory PCa. Further data from testing of androgen-refractory PCa cells and tissues are needed to confirm this hypothesis, however.

## 7. Conclusions

Inevitably, many questions remain unanswered with respect to the various roles played by SUMO pathway members in PCa development and progression. For instance, does the expression of one or multiple SUMOs and SENPs fluctuate with the onset of a given carcinoma, or do specific combinations of SUMOs and SENPs alterations, regarding their function and/or localization, drive the initiation and progression of cancer? It is also difficult to tell yet whether changes in the level of SUMOylation of given transcriptional regulators persist throughout the different stages of PCa. It is possible that both SUMO conjugation and deconjugation are critical for PCa evolution. For example, one arm may be favored at an early stage to initiate tumor growth, while the other arm may be favored at more advanced stages for cancer metastasis [63].

The potential use of elevated SENP1 or/and SENP3 levels in the prostate gland as prognostic markers could possibly identify individuals with an increased risk of developing PCa. In addition, assessment of their levels in PCa samples might serve as diagnostic markers for enhanced angiogenesis and, consequently, more aggressive carcinomas. Additional studies using tissue arrays to validate the use of either SENP1 or SENP3 as biomarkers for PCa are warranted.

Attempts to target the deSUMOylation process by developing specific inhibitors for the family of SENPs are being made, although no pharmacologic agent is currently available. The first report of a successful design of SENP1 inhibitors came only recently, and involves a series of SENP1 inhibitors based on a benzodiazepine scaffold which showed inhibitory activity as high as IC50 = 9.2 μM. The compounds with the best SENP1 inhibitory activity were tested against PCa cells (PC-3) to evaluate their ability to inhibit cancer cell growth *in vitro*, with IC50 values as low as 13.0 μM detected [65]. Hopefully, progression of a selective inhibitor of either SENP1 or SENP3 to *in vivo* testing as well as in the setting of a dedicated clinical trial might lead to an effective therapeutic approach to restoring balance to the aberrant SUMO pathway of PCa patients.
